# Characterization and Function of the Interaction of Angiogenin With Alpha-Actinin 2

**DOI:** 10.3389/fmolb.2022.837971

**Published:** 2022-04-08

**Authors:** Chunhua Weng, Haojie Dong, Jiajia Mao, Xiabing Lang, Jianghua Chen

**Affiliations:** ^1^ Kidney Disease Center, The First Affiliated Hospital, Zhejiang University School of Medicine, Hangzhou, China; ^2^ Key Laboratory of Kidney Disease Prevention and Control Technology, Hangzhou, China; ^3^ National Key Clinical Department of Kidney Diseases, Hangzhou, China; ^4^ Institute of Nephrology, Zhejiang University, Hangzhou, China; ^5^ Zhejiang Clinical Research Center of Kidney and Urinary System Disease, Hangzhou, China; ^6^ Department of Hematological Malignancies Translational Science, Beckman Research Institute, City of Hope Medical Center, Duarte, CA, United States

**Keywords:** angiogenin, alpha-actinin 2, protein–protein interaction, tumor growth, cell proliferation, cell migration, yeast two-hybrid

## Abstract

Angiogenin (ANG) is the first human tumor-derived angiogenic protein, which can promote angiogenesis and tumor growth. In a previous study, we identified alpha-actinin 2 (ACTN2), a cytoskeletal protein, as a direct interacting protein with angiogenin. However, the interaction between ANG and ACTN2 was not characterized in detail, which may provide information on the molecular mechanisms of ANG functions. In this study, we mapped the accurate binding domain and sites in ANG and ACTN2, respectively. In ANG, the residues from 83 to 105 are the smallest motif that can bind to ACTN2. We then use site mutation analysis to identify the precise binding sites of ANG in the interaction and found that the 101st residue arginine (R101) represents the critical residue involved in the ANG–ACTN2 interaction. In ACTN2, the residues from 383 to 632, containing two spectrin domains in the middle of the rod structure of ACTN2, play an important role in the interaction. Furthermore, we validated the interaction of ACTN2-383–632 to ANG by glutathione-S-transferase (GST) pull-down assay. In functional analysis, overexpressed ACTN2-383–632 could impair tumor cell motility observably, including cell migration and invasion. Meanwhile, ACTN2-383–632 overexpression inhibited tumor cell proliferation and survival as well. These data suggest that an excess expression of ACTN2 segment ACTN2-383–632 can inhibit tumor cell motility and proliferation by interfering with the interaction between ANG and ACTN2, which provides a potential mechanism of ANG action in tumor growth and metastasis.

## Introduction

Angiogenin (ANG), a secreted 14-kDa protein, was isolated originally from the conditioned medium of human colon adenocarcinoma HT-29 cells based on its angiogenic activity (Fett et al., 1985). As the first human tumor-derived angiogenic protein, ANG serves as an important regulator both in angiogenesis and tumorigenesis (Gao and Xu, 2008). ANG not only stimulates angiogenesis by activating vessel endothelial cells with a wide range of cellular responses including cell proliferation, migration, invasion, adhesion, and formation of tubular structures but also promotes tumor cell proliferation and vascularization to induce tumor metastasis (Gao and Xu, 2008; Weng et al., 2012). Functional blocking of ANG with antibodies, enzymatic inhibitors, nuclear translocation inhibitors, siRNAs, or nanofibers could prevent tumor angiogenesis and progression and result in targeted cancer therapy (Gao and Xu, 2008; Ibaragi et al., 2009; Li et al., 2020a). Moreover, ANG and its cleavage productions of tRNA, which are called tRNA-derived small RNAs (tsRNA), are both estimated as biomarkers for cancers (Jin et al., 2021; Yu et al., 2021). However, the mechanism of ANG action is not fully clarified.

Protein–protein interactions involved in various biological processes are a hot issue in the protein research field. The ANG binding proteins can be classified in several aspects according to their functions when binding to ANG, including cell motility, proliferation, and apoptosis (Sheng and Xu, 2016). To facilitate cell migration and invasion, extracellular ANG activates plasminogen and matrix metalloproteinase system to promote basement membrane degradation (Hu et al., 1994; Raghu et al., 2010; Dutta et al., 2014), which interacts with several proteins including cell-surface actin (Hu et al., 1993; Pyatibratov et al., 2010; Pyatibratov and Kostyukova, 2012), urokinase plasminogen activator receptor (uPAR), and the plasminogen receptors, A2 and S100-A10 (Dutta et al., 2014). Through receptor-mediated endocytosis, secreted ANG is internalized and undergoes differential subcellular localization in different types of target cells and conditions. Plexin-B2 (PLXNB2) is the essential receptor for ANG binding on the cell surface of target cells (Yu et al., 2017). In the cytoplasm, most of the ANG is kept inactive *via* interaction with human placental ribonuclease inhibitor (RNH1). When stress occurs, the cytoplasmic concentration of active ANG increases, coming from ANG–RNH1 complex dissociation, nuclear ANG translocation to the cytoplasm, and ANG transcription induction (Lyons et al., 2017).

The active ANG in the cytoplasm cleaves tRNAs to form tRNA-derived stress-induced small RNA (tiRNA) in response to stress, thus enhancing cell survival and anti-apoptosis by reprogramming protein synthesis through suppressing global protein translation to save anabolic energy. Under growth conditions, ANG in the nucleus stimulates rRNA transcriptions to promote cell growth and proliferation through interacting with its partners, including follistatin (Gao et al., 2007; Gao et al., 2010), phospholipid scramblase 1 (Zhu et al., 2013), and histone H3 (Sheng et al., 2014). In addition, nuclear ANG binds to p53 and Mdm2, which results in the ubiquitination of p53 (Sadagopan et al., 2012). Cytoplasmic ANG interacts with cytoskeletal proteins, including *α*-actinin 4, non-muscle myosin 9, and *ß*-actin, to optimize focal adhesion formation and stress fiber assembly to promote cell migration (Wei et al., 2011). A critical nuclear factor, proliferating cell nuclear antigen (PCNA), was also found to interact with ANG in the cytoplasm. However, the biological function of this interaction was unknown (Chatzileontiadou et al., 2017).

Among these binding partners, RNH1 and PLXNB2 are two critical proteins interacting with ANG. RNH1, a 50-kDa leucine-rich repeat protein, was identified as the first ANG-binding protein (Shapiro and Vallee, 1987). The interaction between ANG and RNH1 is exceedingly tight with a large contact surface of binding interface, which consists of the C-terminal residues 434–460 of RNH1 and the ribonucleolytic active center in ANG (Papageorgiou et al., 1997). RNH1 binding suppresses both enzymatic and angiogenic actions of ANG (Shapiro and Vallee, 1987). PLXNB2, the 170-kDa putative receptor for ANG on target cell surface as previously reported (Hu et al., 1997), has been identified by Yu et al. (2017) recently. The ANG binding site on PLXNB2 locates at the amino acid residues of 424–441, with a sequence of GTSSEYDSILVEINKRVK. The binding site of PLXNB2 in ANG is a loop region with a sequence of 60-KNGNPHREN-68, which was identified as the receptor binding site, one of the conserved domains in ANG (Sheng and Xu, 2016; Yu et al., 2017). PLXNB2 is the functional receptor of ANG, mediating ANG endocytosis and appropriate subcellular localization in diverse target cells to implement the capabilities of ANG, including cell survival, cell growth, and regenerative activity (Yu et al., 2017). The ANG/PLXNB2 axis has been demonstrated to play pivotal roles in ANG functions in diverse physiologic and pathologic processes (Yu et al., 2017; Bai et al., 2020; Li et al., 2020b; Liu et al., 2021).

In our previous study, we screened a human heart cDNA library with a yeast two-hybrid (Y2H) assay by using ANG as the bait to find out the putative interacting proteins of ANG and to explore the mechanisms of ANG action by clarifying its protein interaction network and their biological functions. Alpha-actinin 2 (ACTN2), a cytoskeletal protein, was identified as a direct interacting protein with ANG (Hu et al., 2005). Their interaction was confirmed *in vitro* and *in vivo* by the methods of His-tagged protein pulled down assay, co-immunoprecipitation, and fluorescence resonance energy transfer analysis (Hu et al., 2005). However, the interaction between ANG and ACTN2 was not characterized in detail, which may provide information on the molecular mechanisms of ANG functions. Thus, in the present study, we employed Y2H and glutathione-S-transferase (GST) pull-down assay to further identify the binding domains and sites of these two proteins, respectively, and evaluated the function of ANG/ACTN2 interaction in tumor cell proliferation and motility.

## Results

### Identification of Binding Sites in ANG

**FIGURE 1:**
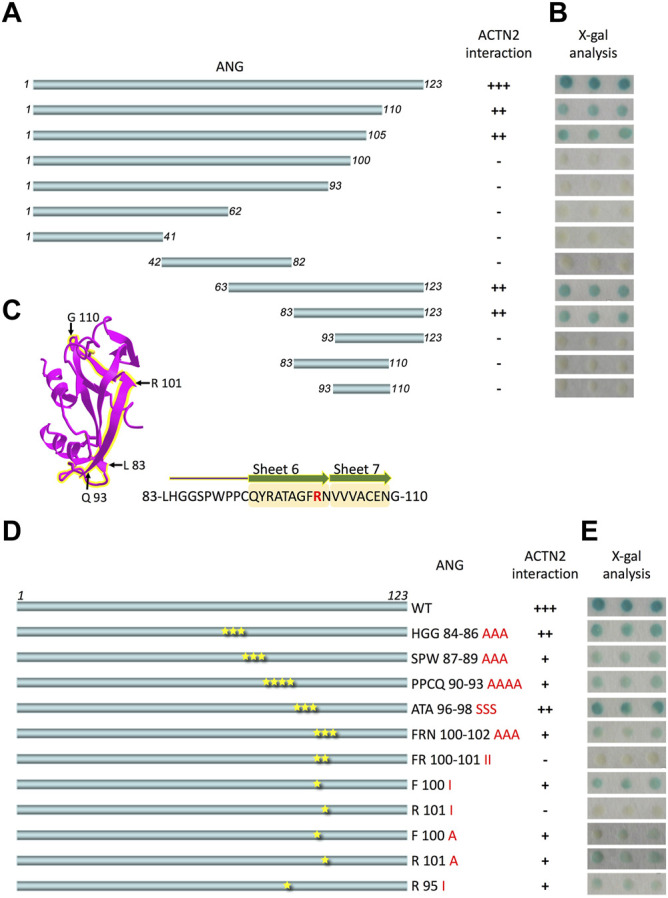
ANG-101st residue arginine (R101) is the critical binding site for alpha-actinin 2 (ACTN2). **(A)** Schematic presentation of the wild-type ANG and deletion constructs used to transform the yeast cells. **(B)** X-gal assay shows the interaction activity of each ANG segment with ACTN2. The pDBLeu constructs containing a different segment of ANG were co-transfected with the plasmid pPC86-ACTN2 into MaV203 yeast cells and examined by the X-gal assay to evaluate the activity of lacZ reporter gene activation. **(C)** Schematic presentation of ANG crystal structure, and the sequence of 83–110 is highlighted in yellow. The sequence of ANG 83–110 contains sheets 6 and 7 mark in yellow shadow and R101 in red. **(D)** Schematic presentation of ANG mutant constructs used to transform the yeast cells. **(E)** X-gal assays show the interaction activity of each ANG mutant with ACTN2. The pDBLeu constructs containing different mutants of ANG were co-transfected with the plasmid pPC86-ACTN2 into MaV203 yeast cells and examined by the X-gal assay to evaluate the activity of lacZ reporter gene activation. All the experiments were carried out more than three times.

ANG is a single-chain protein with 123 amino acid residues. To map the binding motifs or sites for ACTN2 efficiently, the ANG sequence was divided simultaneously into halves or trisections, which were N-terminal half containing residues 1–62 and C-terminal half containing residues 63–123 or segments of residues 1–41, 42–82, and 83–123 (Figure 1A). All of these ANG segments were sent for interaction examination in Y2H assay with ACTN2. The results showed that the C-terminal half and the segment of residues 83–123 of ANG could interact with ACTN2 (Figure 1B), suggesting that the C-terminal of ANG plays an important role in ANG/ACTN2 interaction. To target the binding motif precisely, we made further deletions on the segment of residues 83–123 at either or both sides, to create shorter segments of residues 83–110, 93–123, and 93–110 (Figure 1A) and to examine the interacting ability to ACTN2. The results showed that none of these three segments of ANG bind to ACTN2 in the Y2H assay (Figure 1B). These results demonstrate that the ANG segment of residues 83–123 is the smallest domain that can interact with ACTN2 independently in the Y2H assay.

To further identify the binding site in ANG interacting with ACTN2, we made a series of deletion constructs from the C-terminal of ANG, including the segments of residues 1–110, 1–105, 1–100, and 1–93 (Figure 1A). The results showed that the segments of residues 1–110 and 1–105 have a strong interacting ability with ACTN2 (Figure 1B). Thus, the segments of residues 1–105 and 83–123 were the shortest deletion constructs from the C-terminal or N-terminal, respectively, suggesting that the binding sites for ACTN2 should locate in the sequence of 83-LHGGSPWPPCQYRATAGFRNVVV-105. This motif of residues 83–105 includes a coil and an integrated S6 *ß*-sheet and a partial of S7 *ß*-sheet in the crystal structure of ANG (NCBI Structure Database, MMDB ID: 142416) (Figure 1C). Then, we made a series of site mutations among the residues 83–105 to identify the precise binding site(s) in ANG (Figure 1D). As the results show, when the 101st residue arginine (R101) mutated to isoleucine (I), the interaction ability of ANG to ACTN2 was totally vanished, indicating that R101 of ANG plays a pivotal role in the interaction of ANG/ACTN2 (Figure 1E).

### ANG Binds to the Middle Spectrin Domains of ACTN2

**FIGURE 2:**
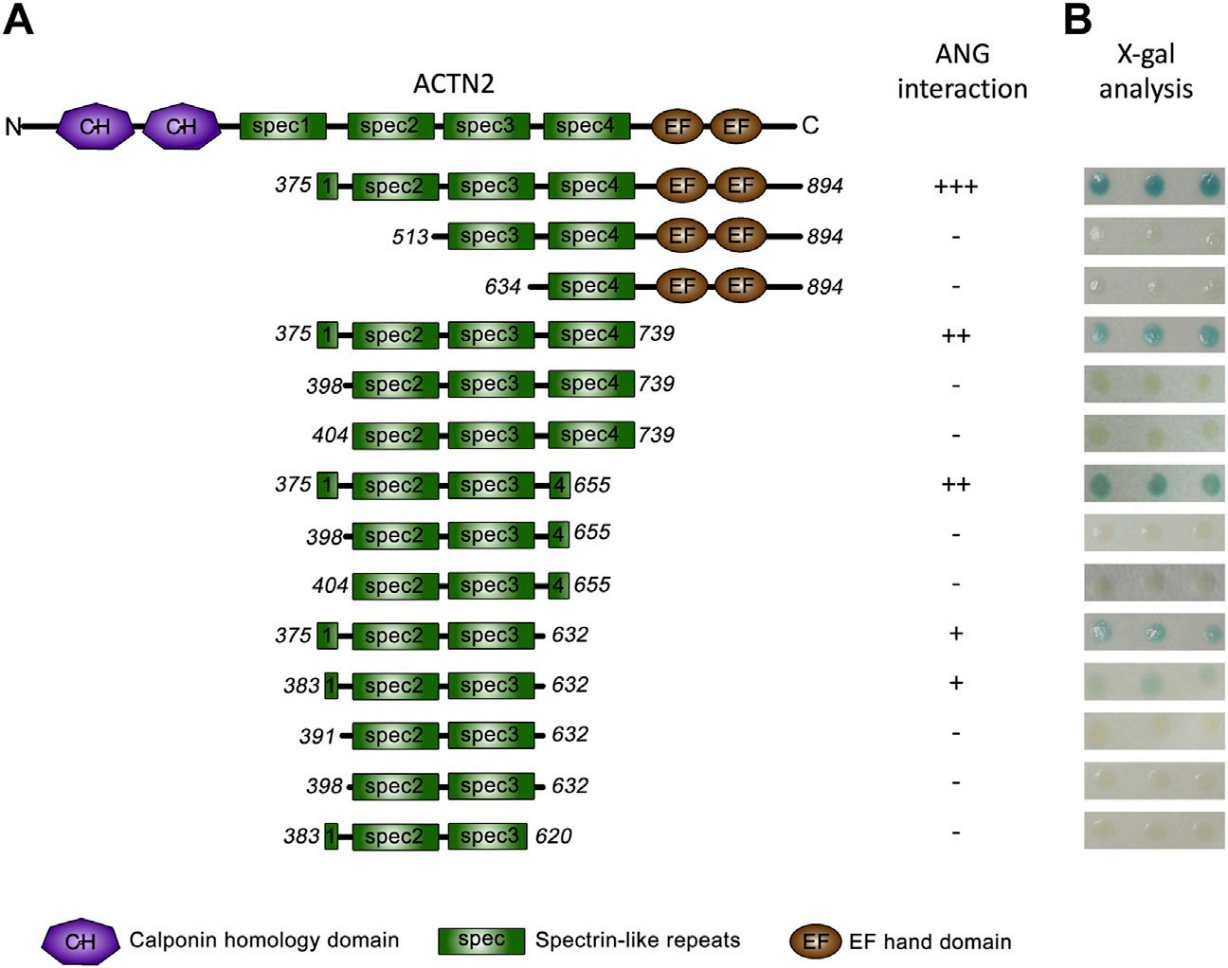
ANG binds to ACTN2 at the middle spectrin domains. **(A)** Schematic presentation of the wild-type ACTN2 domains and various deletion constructs used to transform yeast cells. **(B)** X-gal assays show the interaction activity of each ACTN2 segment with ANG. The pPC86 constructs containing a different segment of ACTN2 were co-transfected with the bait plasmid pDBLeu-ANG into MaV203 yeast cells and examined by the X-gal assay to evaluate the activity of lacZ reporter gene activation. All the experiments were carried out more than three times.

We then focused on ACTN2 to analyze the interacting domains for ANG. As we previously reported, among the ACTN2 cDNA screened by yeast two-hybrid assay, the shortest segment of ACTN2 was the segment of residues from 375 to 894 (Hu et al., 2005), which contains a part of the first spectrin domain, three integrated spectrin domains, and two EF-hand domains at the C-terminal (Figure 2A). We constructed a series of deletions from both sides of the segment ACTN2-375–894 to detect the ability of ANG interaction. As the results showed that when one or two-spectrin domains on the N-terminal of the ACTN2-375–894 were deleted, the interaction ability to ANG was lost. Whereas, deletion of the two EF-hand domains at the C-terminal almost did not affect the interaction of ANG/ACTN2, as the results showed that the segment of ACTN2-375–739 without C-terminal EFs still has a strong binding ability to ANG (Figures 2A,B). Next, we made several deletion constructs based on ACTN2-375–739 and detected their interaction with ANG. We found that the smallest segment binding to ANG independently in Y2H assay was ACTN2-383–632, which contains a small part of the first spectrin domain and the integrated second and third spectrin domains. Any of the segments shorten by either side of ACTN2-383–632, such as ACTN2-391–632, -398–632, or -383–620, could not interact with ANG (Figure 2A,B). In addition, the small part of the first spectrin domain, which starts from the 383rd residue of ACTN2, is necessary for ANG binding. If the segments start from the residues after the 383rd residue, such as the segments of ACTN2-398–655 or -404–655 and the segments of ACTN2-398–739 or -404–739, the binding ability to ANG was lost, even though there was an additional small partial or integrated domain of the fourth spectrin domain at the C-terminal. Our results demonstrated that ACTN2-383–632 at the central structure of ACTN2 binds to ANG.

### ACTN2-383–632 Segment Binds to ANG *In Vitro*

**FIGURE 3:**
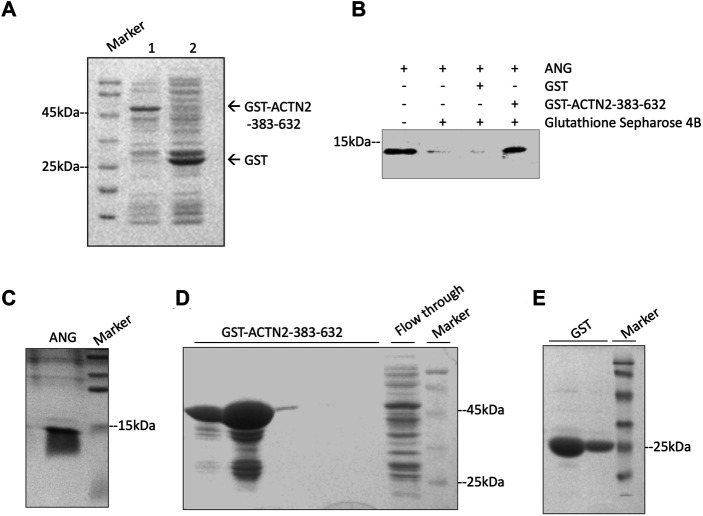
ACTN2-383–632 interacts with ANG *in vitro*. **(A)** Recombinant glutathione-S-transferase (GST)-tagged ACTN2-383–632 and GST were expressed successfully in *E. coli*. Lane 1, GST-ACTN2-383–632 protein in the bacterial supernatant after isopropyl-1-thiob-d-galactopyranoside (IPTG) induction; lane 2, GST-tagged protein in the bacterial supernatant after IPTG induction. **(B)** ANG was pulled down by GST-ACTN2-383–632 specifically. Purified recombinant ANG was mixed with GST or GST-ACTN2-383–632 protein purified with Glutathione Sepharose 4B, and the pull-down products were detected by immunoblotting. **(C–E)** SDS-PAGE analysis of purified ANG, GST-ACTN2-383-632, and GST protein stained with coomassie bright blue (CBB).

To confirm the interaction of ACTN2-383–632 with ANG, we employed a GST pull-down experiment to analyze their binding ability *in vitro*. In the GST pull-down experiment, recombinant GST-tagged ACTN2-383–632 protein or GST protein was induced by isopropyl-1-thiob-d-galactopyranoside (IPTG) and expressed (Figure 3A), purified by Glutathione Sepharose 4B from bacterial supernatant, and then incubated with recombinant ANG protein in a binding reaction. The final pull-down products by Glutathione Sepharose 4B were washed and collected for immunoblotting with an anti-ANG polyclonal antibody. The results showed that ANG was detected in the pull-down products with GST-tagged ACTN2-383–632, but not in the GST control reaction (Figure 3B). These results indicate that ACTN2-383–632 can interact with ANG specifically and directly *in vitro*.

### ACTN2-383–632 Segment Inhibits Tumor Cell Motility and Proliferation

**FIGURE 4:**
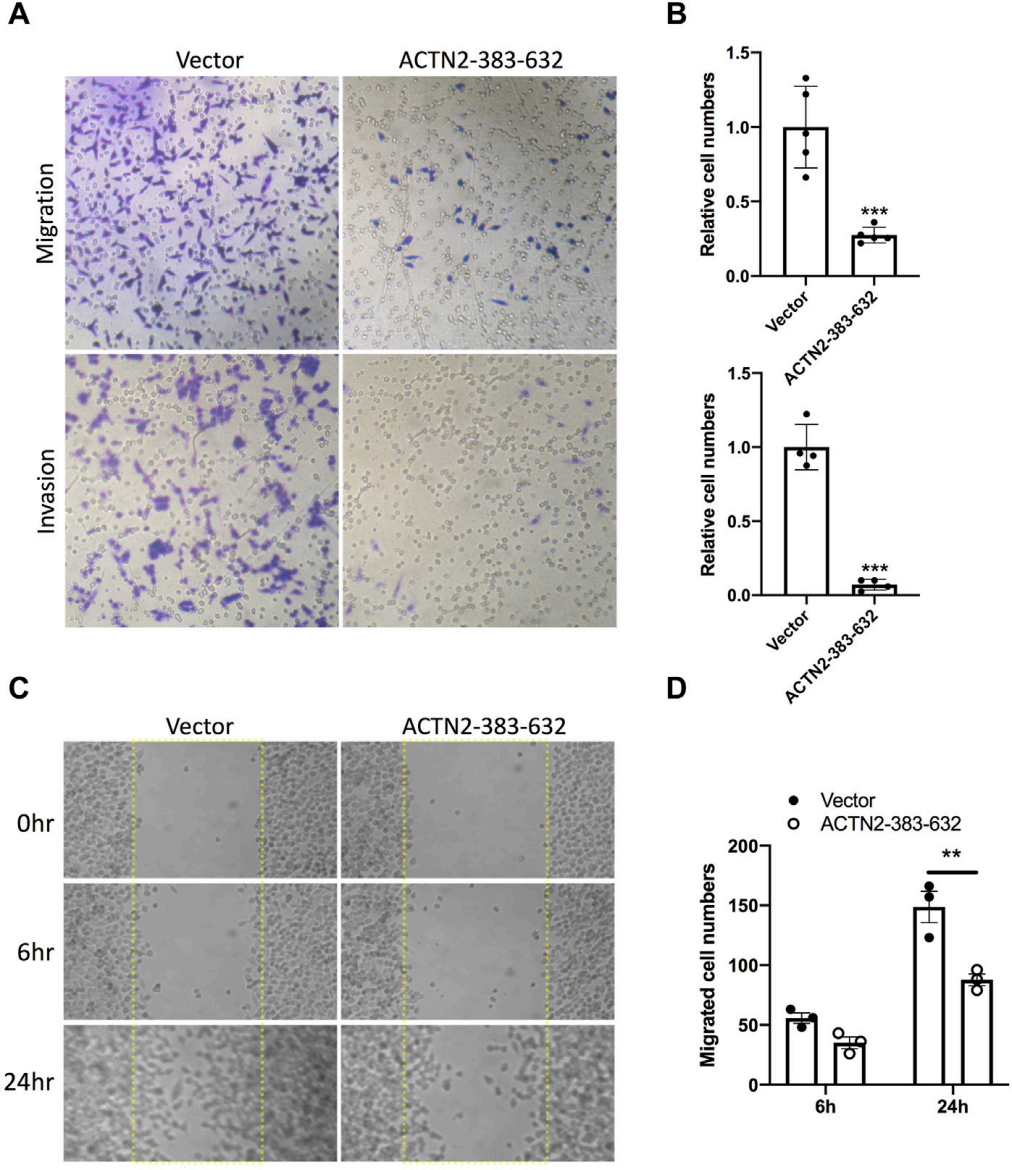
ACTN2-383–632 segment inhibits tumor cell migration and invasion. **(A–B)** Representative images and quantification statistics of transwell migration assay and Matrigel-coated transwell invasion assay. Hela cells overexpressing ACTN2-383–632 (empty vector as a control) were seeded in chambers with 5% serum medium as a migratory stimulant. Cells that migrated through the chamber were quantified to evaluate the migration or invasion activity. **(C–D)** Representative images and quantification statistics of wound healing assay. Hela cells transfected with ACTN2-383–632 overexpressing plasmid or vector were grown to a confluent monolayer for a wound healing assay. Cells migrated into the scratched wound area (dashed line delineated) were quantified to evaluate the migration activity. All the experiments were carried out more than three times and presented as means ± SD, ***p < 0.005, **p < 0.01.

**FIGURE 5:**
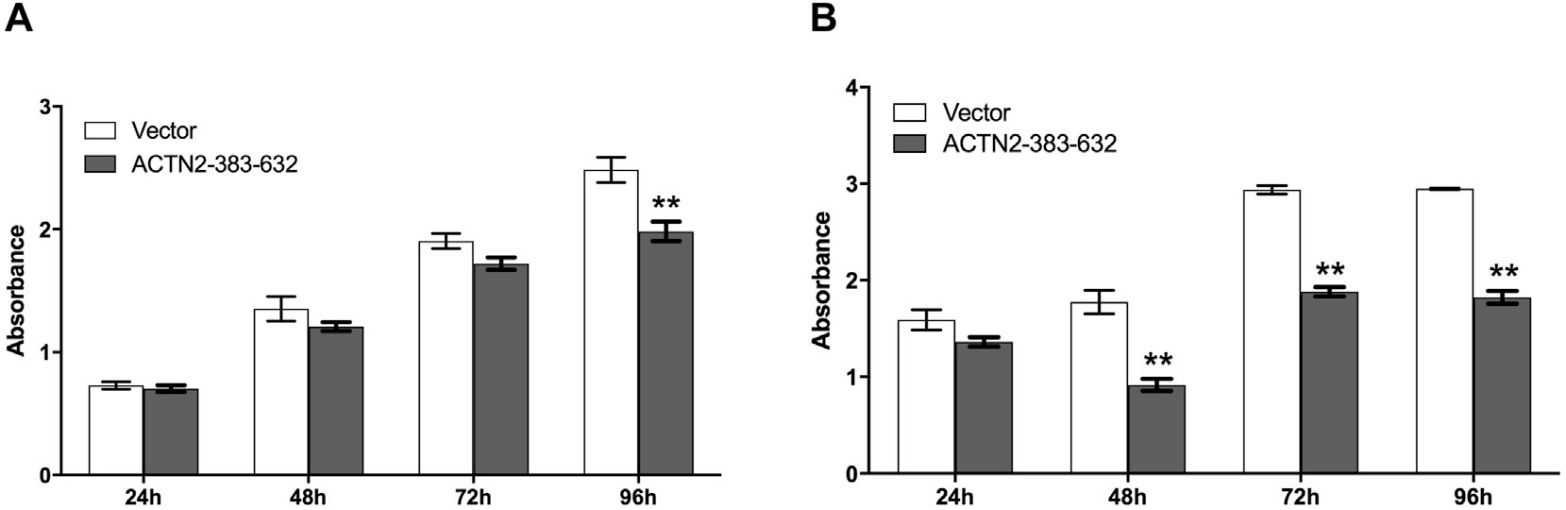
ACTN2-383–632 segment inhibits tumor cell proliferation and survival. **(A)** Hela cells were transfected with ACTN2-383–632 expression construct for 24 h, and empty vector transfection served as a control. The cells were subjected to examination with the CCK-8 kit at 24, 48, 72, and 96 h after reseeding. **(B)** Hela cells were transfected with ACTN2-383–632 expression construct for 24 h, and empty vector transfection served as a control. The cells were cultured in a fetal bovine serum (FBS)-free medium after reseeding overnight and subjected to examination with the CCK-8 kit at 24, 48, 72, and 96 h. All the experiments were carried out more than three times and presented as means ± SD, **p < 0.01.

To explore the functions of ANG–ACTN2 interaction, we analyzed cell migration and invasion, as well as cell proliferation and survival with tumor cells, by overexpressing the segment of ACTN2-383–632. In cell migration assays, both in transwell assay and wound healing assay, ACTN2-383–632 segment overexpression inhibited Hela cell migration observably (Figures 4A–D). Meanwhile, we employed Matrigel-coated transwell invasion assay to find whether tumor cell invasion could also be inhibited by ACTN2-383–632 segment overexpression (Figures 4A, B). On the other hand, we analyzed the cell proliferation and survival ability of Hela cells to examine the functions of the ACTN2-383–632 segment. The results showed that ACTN2-383–632 segment overexpression not only reduced cell proliferation (Figure 5A) but also inhibited cell survival significantly (Figure 5B). These results suggested that overloaded ACTN2-383–632 segment in the cytoplasm may interfere with the interaction of ANG to wild-type ACTN2, as well as block the functions and secretion of cytoplasmic ANG, which affects tumor cell motility and proliferation and thus tumor growth or metastasis.

## Discussion

ACTN2 is a protein that interacts with ANG in the cytoplasm we identified previously (Hu et al., 2005). However, the characterization and function of the interaction of ANG with ACTN2 have not yet been fully elucidated. In this study, we identified the binding domains and sites in ANG and ACTN2 and evaluated the function of this interaction in tumor cell behaviors.

**FIGURE 6:**
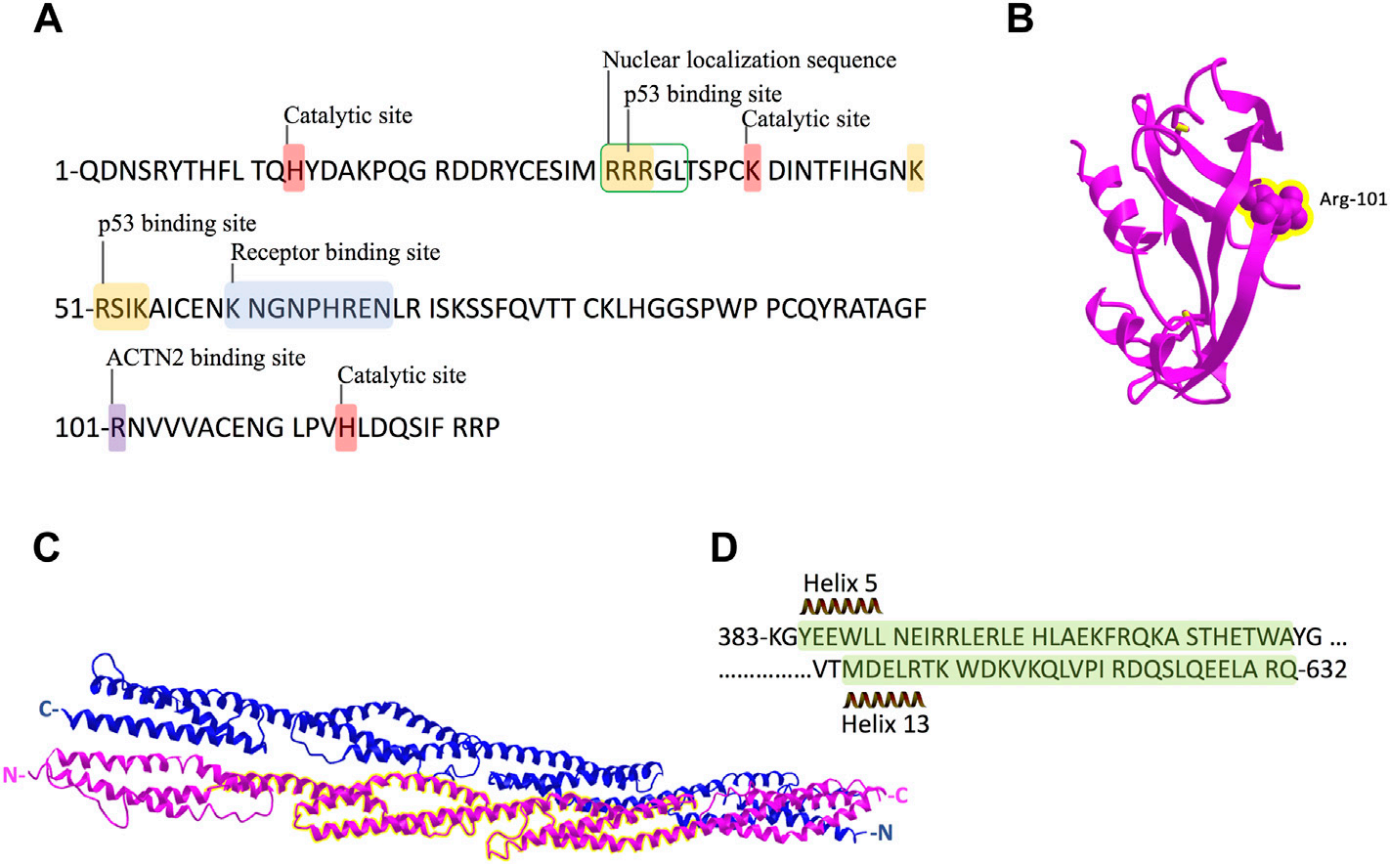
Schematic representation of ANG and ACTN2 protein sequence and structure. **(A)** ANG is a single-chain protein with 123 amino acids and contains several conserved domains: receptor-binding site K60–N68; the catalytic site of H14, K40, and H114; and nuclear localization sequence R31–L35. The binding sites of p53 in ANG are 31R–33R and 50–54K. R101 is the binding site we identified for ACTN2 in this study. **(B)** Crystal structure of ANG with the residue R101 showed in sphere form and highlighted in yellow. **(C)** Crystal structure of the rod domain of *α*-ACTN-2, with the residues 383–632 highlighted in yellow. **(D)** The sequence of ACTN2 383–632 with helix 5 and 13 is marked in green shadow.

ANG, a 14-kDa basic protein, is the fifth member of the RNase A superfamily, which has 33% sequence identity and 65% homology with bovine pancreatic RNase A (Shapiro et al., 1986). ANG has a nuclear localization sequence, a catalytic center, and a receptor binding site (Figure 6A), constituting a special structure to carry out its diverse biological functions (Sheng and Xu, 2016). The receptor binding site of ANG consists of residues from Lys-60 to Asn-68, forming a loop region to interact with actin and PLXNB2 at the cell surface (Hu et al., 1991; Acharya et al., 1994; Yu et al., 2017). Mutations of R66A and N68D at this receptor binding site of ANG could abolish binding to PLXNB2 (Yu et al., 2017). The catalytic center (His-13, Lys-40, and His-114), the same general catalytic residues as in bovine pancreatic RNase A, cleaves the phosphodiester bond of ribonucleic acid (Palmer et al., 1986; Shapiro et al., 1986). Although its ribonucleolytic activity is only 10^–5^ to 10^−6^-fold of what is in RNase A, this activity is essential for ANG to perform its various biological functions (Sheng and Xu, 2016). The nuclear localization sequence 31RRRGL35 is responsible for the nucleolar targeting of ANG (Moroianu and Riordan, 1994a; Moroianu and Riordan, 1994b).

In protein–protein interactions, residues from Arg-31 to Arg-33 (31RRR33) and residues from Lys-50 to Lys-54 (50KRSIK54) are two motifs in ANG identified as p53 binding sites (Yeo et al., 2017). These two motifs are positively charged, which could interact with negatively charged residues D48/E51 and E56 in the p53 TAD2 domain (Yeo et al., 2017). In this study, we found that the residue Arg-101(R101) of ANG is the critical binding site to ACTN2, which is not involved in any conserved domains of ANG (Figure 6A). Meanwhile, R101 is a positively charged residue, which locates at the surface of the ANG protein structure (Figure 6B) as shown in the crystal structure in a 3D viewer (NCBI Structure Database, MMDB ID: 142416). In addition, the position of R101 is at the right-angled bulge formed by the connection of the *ß*-sheets S6 and S7 (Figures 1C, 6B), which makes R101 have a higher probability to interact with ACTN2 or other proteins. These data suggest that the interaction between ANG and ACTN2 may be the same as with p53, which is conducted by positive–negative-charged residues. However, the precise binding sites in ACTN2 need to be further identified.

**FIGURE 7:**
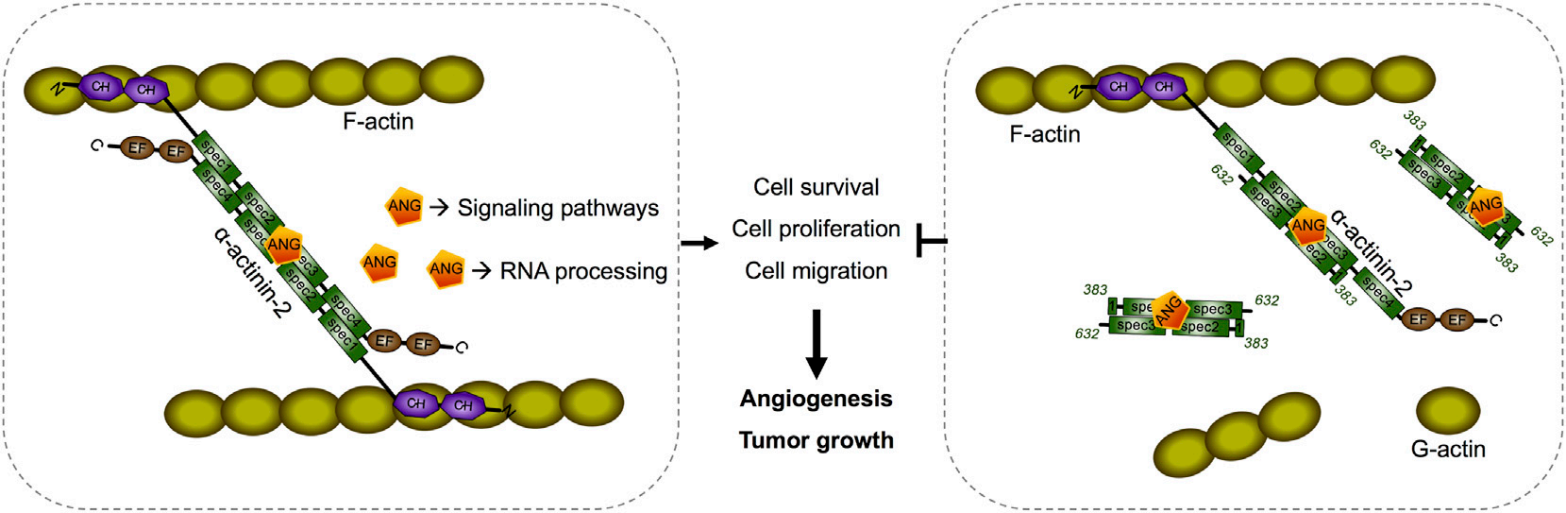
Schematic diagram of a predicted model of segment ACTN2 383–632/ANG interaction and its interference in angiogenesis and tumor growth. The segment ACTN2 383–632 in cell binds to another molecule of ACTN2 383–632 or wild-type ACTN2 to form an anti-parallel structure that interacts with ANG. When the segment ACTN2 383–632 is overloaded, the functions of ANG and ACTN2 are disrupted, such as cell proliferation, survival, and migration, which results in inhibition of angiogenesis or tumor growth.

ACTN2 belongs to the proteins of the spectrin superfamily, which plays key roles in the actin filament crosslinks and the actin cytoskeleton assembling to maintain cell structural integrity, as well as cell signaling in various cell types (Ribeiro et al., 2014). ACTN2 is comprised of an actin-binding domain (ABD), which consists of the two calponin homology domains (CH1 and CH2) at the N-terminal, a pair of EF-hand domains (EFs) at the C-terminal, and four spectrin-like repeats in the central domain, combining an anti-parallel homodimer with another molecular of ACTN2 (Ribeiro et al., 2014). The spectrin region is also called the rod domain, as it appears to have a cylindrical shape (NCBI Structure Database, MMDB ID: 16612) (Figure 6C), which provides interaction sites for multiple signaling and structural proteins, including ALP (actinin-associated LIM protein), myotilin, PKN, titin, zyxin, the NMDA receptor, and FATZ (Hu et al., 2005). The segment of residues 383–632 of ACTN2 showed that interaction with ANG was also located in this rod domain (Figures 6C,D), suggesting that ANG is one of the components that assembles a multiprotein complex with ACTN2 involved in the cell motility such as cell migration and invasion. In addition, the segment of residues 383–632 of ACTN2 may also be the smallest component to form an anti-parallel homodimer to provide the binding motif for ANG or other proteins. The segment ACTN2 383–632 in cell binds to another molecule of ACTN2 383–632 or wild-type ACTN2 to form an anti-parallel structure that interacts with ANG (Figure 7). When the segment ACTN2 383–632 is overloaded, the functions of ANG and ACTN2 would be disrupted, such as cell proliferation, survival, and migration, which results in angiogenesis or tumor growth inhibition. We speculate that ACTN2 383–632 without interaction with ANG could also disrupt the functions of cell motility. Thus, overexpressing ACTN2 383–632 in ANG knockout cells is necessary to access the effects of ACTN2 383–632 in future work.

Interestingly, actin is an important binding partner for both ANG and ACTN2. Actin is a ubiquitous protein that plays a key role in cell motility, cell structure, and contraction in both muscle and non-muscle cells. Although actin is more commonly known as an intracellular protein, it was also found on the external surface of endothelial cells, fibroblasts, monocytes, lymphocytes, and smooth muscle cells (Pyatibratov and Kostyukova, 2012). ANG was found to interact with actin on the cell surface of endothelial cells by the receptor binding site, which is conserved among angiogenin from various species (Hu et al., 1991; Hu and Riordan, 1993; Pyatibratov and Kostyukova, 2012). The cell surface actin of endothelial cells is considered to be an extracellular matrix (ECM)-actin *in vivo*; thus, ANG binds to ECM-actin, which could result in activation of a cell-associated protease system that leads to cell detachment from the ECM followed by cell migration, proliferation, and differentiation into microvessels (Hu and Riordan, 1993; Pyatibratov and Kostyukova, 2012). In the cytoplasm, ANG can interact with intracellular actin indirectly through cytosolic ACTN2 (Hu et al., 2005). These suggest that ANG promotes cell motility and proliferation, which are both critical in processes in tumor growth and development, through extracellular as well as intracellular pathways involving the actions of actin.

In conclusion, our data demonstrate that ACTN2 binds to the motif of residues 83–105 of ANG, and R101 is the critical binding site of the interaction. The residues 383–632 of ACTN2 are the binding region for ANG. Overloaded ACTN2 383–632 can interfere with tumor cell migration, invasion, cell proliferation, and survival. These findings may partly account for the molecular mechanism underlining ANG-induced angiogenesis, tumor growth, and metastasis, providing a potential therapeutic target for tumors.

## Materials and Methods

### Plasmid Construct

The Human ACTN2 gene was screened by yeast two-hybrid assay with human heart cDNA library. ACTN2 segments with different lengths were obtained by PCR amplifications and inserted into pPC86, pGEX-2T, or pcDNA3.1(+) vectors. The human ANG gene and its deletions were also obtained by PCR amplification and inserted into the pDBLeu vector. The site-directed ANG mutants were obtained by the nest-PCR method with mega-primer PCR mutagenesis. All primers used were synthesized in Sangong (Sangong, Shanghai, China). Primer sequences are listed in Supplementary Table S1. Each construct was verified by sequencing.

### Yeast Two-Hybrid Assay

The ProQuest Two-Hybrid System (Gibco) was employed to map the binding domain of ACTN2 and ANG. As previously described (Hu et al., 2005), pairs of plasmids containing a different segment of ACTN2 or ANG were co-transfected into yeast cells. X-gal analysis was used to evaluate the interactions. Control strains A, B, C, D, and E (Gibco, United States) that contain plasmid pairs that express fusion proteins with a spectrum of interaction strengths, including none, weak, moderately strong, strong, and very strong, respectively, were used to assess the interaction strength between ACTN2 and ANG. Each co-transformation was conducted three times to confirm the results.

### GST Pull-Down Assay

GST-tagged ACTN2 protein was expressed by inserting ACTN2-383–632 DNA segments into the pGEX-2T vector to create the plasmid pGEX-2T-ACTN2-383–632. The plasmid was transformed into *Escherichia coli* strain BL21 (DE3) and induced by IPTG to express GST fusion protein. As the GST and GST-ACTN2-383–632 proteins were expressed in the supernatant of bacterial lysate, they were then purified with GSTrap-FF affinity chromatography (GE Healthcare, Uppsala, Sweden) following the manufacturer’s instruction. Then, 50 μl Glutathione Sepharose 4B slurries were pre-washed and incubated with 20 μg of purified ACTN2 or 10 μg GST proteinm respectively. After washing three times, the purified proteins were incubated respectively with 5 μg recombinant ANG with a concentration of about 2 μM in binding buffer (50 mM Tris, 50 mM NaCl, 2 mM EDTA, 10% glycerol, 0.5% NP-40, 25 mM HEPES, and 1 mM PMSF) at 4°C for 1 h with gentle rotation. After incubation, the resins were washed with binding buffer three times, and the bound proteins were subjected to western blotting and detected with an anti-ANG antibody 26-2F.

### Cell Culture and Transfection

Hela cells were cultured in Dulbecco’s modified Eagle’s medium (GIBCO) supplemented with 10% fetal bovine serum (Hyclone) and incubated in an incubator with a 5% CO_2_ humidified atmosphere at 37°C. Transfections were performed with Lipofectamine 2000 reagent (Invitrogen) following the instructions of the manufacturer.

### Cell Migration and Invasion Assay

Cell migration assay was performed using a 24-well transwell plate with a Transwell Boyden Chamber with a membrane pore size of 8 μm (Millipore). After 24-h transfection, cells were collected and re-suspended in serum-free culture medium at a concentration of 2.5 × 10^5^ cells/ml, and 200 μl of the cell suspension (5 × 10^4^ cells) was added into each upper compartment of the chamber. Then, 5% fetal bovine serum (FBS) was added to the lower compartment. After 6-h incubation, the membrane-containing cells were fixed with 4% paraformaldehyde for 15 min at 4°C and stained with 0.1% crystal violet in 0.1 M borate and 2% ethanol for 15 min at room temperature. Non-migrated cells remaining on the upper surface of the membrane were wiped off with a cotton swab. Migrated cells were quantified by blind counting of five random microscopy fields per well at ×400 magnification. Triplicate studies were performed for each experimental condition. For invasion assay, Matrigel (BD Biosciences)-coated transwell chambers were used to incubate cells, and the following steps were performed for the migration assay.

### Wound Healing Assay

Cells were seeded in a six-well plate with marker lines at the bottom, after transfection with ACTN2 expression plasmids or empty vectors. A “scratch” in a monolayer of confluent cells was generated with a P200 pipette tip, and the scraped-off cells were washed away by PBS. The cells were cultured in FBS-free medium thereafter. Migrated cells were monitored by taking pictures at the same view (crosses of scratch and marker line) at 0, 6, and 24 h; counting; and analyzing with statistics.

### Cell Proliferation and Survival Assay

Cell proliferation was carried out using the Cell Counting Kit (Dojindo Molecular Technologies, Kumamoto, Japan). Cells were collected at 24 h after transfection with plasmids, reseeded in 96-well plates at a density of 1,000 cells per well, and then incubated overnight. Cell numbers were measured at 24, 48, 72, and 96 h with the CCK-8 kit. For cell survival assay, cells were cultured in FBS-free medium, and cell numbers were measured at 24, 48, 72, and 96 h with the CCK-8 kit.

### Statistics

All data are presented as means ± SD. The statistical analysis was performed with the unpaired *t*-tests for comparison of two groups. One-way ANOVA was used for comparison among multiple groups. *p* < 0.05 was considered to be significant.

## Data Availability

The original contributions presented in the study are included in the article/Supplementary Material; further inquiries can be directed to the corresponding authors.

## References

[B1] Acharya K. R. Shapiro R. Allen S. C. Riordan J. F. Vallee B. L. (1994). Crystal Structure of Human Angiogenin Reveals the Structural Basis for its Functional Divergence from Ribonuclease. Proc. Natl. Acad. Sci. 91 (8), 2915–2919. 10.1073/pnas.91.8.2915 8159679 PMC43485

[B2] Bai R. Sun D. Chen M. Shi X. Luo L. Yao Z. (2020). Myeloid Cells Protect Intestinal Epithelial Barrier Integrity through the Angiogenin/plexin-B2 axis. EMBO J. 39 (13), e103325. 10.15252/embj.2019103325 32510170 PMC7327495

[B3] Chatzileontiadou D. S. M. Samiotaki M. Alexopoulou A. N. Cotsiki M. Panayotou G. Stamatiadi M. (2017). Proteomic Analysis of Human Angiogenin Interactions Reveals Cytoplasmic PCNA as a Putative Binding Partner. J. Proteome Res. 16 (10), 3606–3622. 10.1021/acs.jproteome.7b00335 28777577

[B4] Dutta S. Bandyopadhyay C. Bottero V. Veettil M. V. Wilson L. Pins M. R. (2014). Angiogenin Interacts with the Plasminogen Activation System at the Cell Surface of Breast Cancer Cells to Regulate Plasmin Formation and Cell Migration. Mol. Oncol. 8 (3), 483–507. 10.1016/j.molonc.2013.12.017 24457100 PMC5528647

[B5] Fett J. W. Strydom D. J. Lobb R. R. Alderman E. M. Bethune J. L. Riordan J. F. (1985). Isolation and Characterization of Angiogenin, an Angiogenic Protein from Human Carcinoma Cells. Biochemistry 24 (20), 5480–5486. 10.1021/bi00341a030 4074709

[B6] Gao X. Hu H. Zhu J. Xu Z. (2007). Identification and Characterization of Follistatin as a Novel Angiogenin-Binding Protein. Febs Lett. 581 (28), 5505–5510. 10.1016/j.febslet.2007.10.059 17991437

[B7] Gao X. Wei S. Lai K. Sheng J. Su J. Zhu J. (2010). Nucleolar Follistatin Promotes Cancer Cell Survival under Glucose-Deprived Conditions through Inhibiting Cellular rRNA Synthesis. J. Biol. Chem. 285 (47), 36857–36864. 10.1074/jbc.m110.144477 20843798 PMC2978615

[B8] Gao X. Xu Z. (2008). Mechanisms of Action of Angiogenin. Acta Biochim. Biophys. Sin (Shanghai) 40 (7), 619–624. 10.1111/j.1745-7270.2008.00442.x 18604453

[B9] Hu G.-f. Riordan J. F. Vallee B. L. (1997). A Putative Angiogenin Receptor in Angiogenin-Responsive Human Endothelial Cells. Proc. Natl. Acad. Sci. 94 (6), 2204–2209. 10.1073/pnas.94.6.2204 9122172 PMC20065

[B10] Hu G. F. Chang S. I. Riordan J. F. Vallee B. L. (1991). An Angiogenin-Binding Protein from Endothelial Cells. Proc. Natl. Acad. Sci. 88 (6), 2227–2231. 10.1073/pnas.88.6.2227 2006162 PMC51203

[B11] Hu G. F. Riordan J. F. (1993). Angiogenin Enhances Actin Acceleration of Plasminogen Activation. Biochem. Biophysical Res. Commun. 197 (2), 682–687. 10.1006/bbrc.1993.2533 8267604

[B12] Hu G. F. Strydom D. J. Fett J. W. Riordan J. F. Vallee B. L. (1993). Actin Is a Binding Protein for Angiogenin. Proc. Natl. Acad. Sci. 90 (4), 1217–1221. 10.1073/pnas.90.4.1217 7679494 PMC45843

[B13] Hu G. Riordan J. F. Vallee B. L. (1994). Angiogenin Promotes Invasiveness of Cultured Endothelial Cells by Stimulation of Cell-Associated Proteolytic Activities. Proc. Natl. Acad. Sci. 91 (25), 12096–12100. 10.1073/pnas.91.25.12096 7991590 PMC45383

[B14] Hu H. Gao X. Sun Y. Zhou J. Yang M. Xu Z. (2005). α-Actinin-2, a Cytoskeletal Protein, Binds to Angiogenin. Biochem. Biophysical Res. Commun. 329 (2), 661–667. 10.1016/j.bbrc.2005.01.158 15737636

[B15] Ibaragi S. Yoshioka N. Li S. Hu M. G. Hirukawa S. Sadow P. M. (2009). Neamine Inhibits Prostate Cancer Growth by Suppressing Angiogenin-Mediated rRNA Transcription. Clin. Cancer Res. 15 (6), 1981–1988. 10.1158/1078-0432.ccr-08-2593 19276260 PMC2670466

[B16] Jin F. F. Yang L. Q. Wang W. X. Yuan N. Zhan S. B. Yang P. (2021). A Novel Class of tsRNA Signatures as Biomarkers for Diagnosis and Prognosis of Pancreatic Cancer. Mol. Cancer 20 (1). 10.1186/s12943-021-01389-5 PMC8285832 34273975

[B17] Li S. Goncalves K. A. Lyu B. Yuan L. Hu G. F. (2020). Chemosensitization of Prostate Cancer Stem Cells in Mice by Angiogenin and Plexin-B2 Inhibitors. Commun. Biol. 3 (1), 26. 10.1038/s42003-020-0750-6 31942000 PMC6962460

[B18] Li Y. Qu X. Cao B. Yang T. Bao Q. Yue H. (2020). Selectively Suppressing Tumor Angiogenesis for Targeted Breast Cancer Therapy by Genetically Engineered Phage. Adv. Mater. 32 (29), e2001260. 10.1002/adma.202001260 32495365

[B19] Liu X. N. Chai Y. Liu G. Q. Su W. P. Guo Q. Y. Lv X. (2021). Osteoclasts Protect Bone Blood Vessels against Senescence through the Angiogenin/plexin-B2 axis. Nat. Commun. 12 (1). 10.1038/s41467-021-22131-1 PMC7987975 33758201

[B20] Lyons S. M. Fay M. M. Akiyama Y. Anderson P. J. Ivanov P. (2017). RNA Biology of Angiogenin: Current State and Perspectives. RNA Biol. 14 (2), 171–178. 10.1080/15476286.2016.1272746 28010172 PMC5324756

[B21] Moroianu J. Riordan J. F. (1994). Identification of the Nucleolar Targeting Signal of Human Angiogenin. Biochem. Biophysical Res. Commun. 203 (3), 1765–1772. 10.1006/bbrc.1994.2391 7945327

[B22] Moroianu J. Riordan J. F. (1994). Nuclear Translocation of Angiogenin in Proliferating Endothelial Cells Is Essential to its Angiogenic Activity. Proc. Natl. Acad. Sci. 91 (5), 1677–1681. 10.1073/pnas.91.5.1677 8127865 PMC43226

[B23] Palmer K. A. Scheraga H. A. Riordan J. F. Vallee B. L. (1986). A Preliminary Three-Dimensional Structure of Angiogenin. Proc. Natl. Acad. Sci. 83 (7), 1965–1969. 10.1073/pnas.83.7.1965 3457369 PMC323210

[B24] Papageorgiou A. C. Shapiro R. Acharya K. R. (1997). Molecular Recognition of Human Angiogenin by Placental Ribonuclease Inhibitor_an X-ray Crystallographic Study at 2.0Aresolution. Embo J. 16 (17), 5162–5177. 10.1093/emboj/16.17.5162 9311977 PMC1170149

[B25] Pyatibratov M. G. Kostyukova A. S. (2012). New Insights into the Role of Angiogenin in Actin Polymerization. Int. Rev. Cel Mol. Biol. 295295, 175–198. 10.1016/b978-0-12-394306-4.00011-3 PMC5828769 22449490

[B26] Pyatibratov M. G. Tolkatchev D. Plamondon J. Xu P. Ni F. Kostyukova A. S. (2010). Binding of Human Angiogenin Inhibits Actin Polymerization. Arch. Biochem. Biophys. 495 (1), 74–81. 10.1016/j.abb.2009.12.024 20045391 PMC2831474

[B27] Raghu H. Lakka S. S. Gondi C. S. Mohanam S. Dinh D. H. Gujrati M. (2010). Suppression of uPA and uPAR Attenuates Angiogenin Mediated Angiogenesis in Endothelial and Glioblastoma Cell Lines. Plos One 5 (8), e12458. 10.1371/journal.pone.0012458 20805979 PMC2929192

[B28] Ribeiro E. d. A. Pinotsis N. Ghisleni A. Salmazo A. Konarev P. V. Kostan J. (2014). The Structure and Regulation of Human Muscle α-Actinin. Cell 159 (6), 1447–1460. 10.1016/j.cell.2014.10.056 25433700 PMC4259493

[B29] Sadagopan S. Veettil M. V. Chakraborty S. Sharma-Walia N. Paudel N. Bottero V. (2012). Angiogenin Functionally Interacts with P53 and Regulates P53-Mediated Apoptosis and Cell Survival. Oncogene 31 (46), 4835–4847. 10.1038/onc.2011.648 22266868 PMC3337890

[B30] Shapiro R. Riordan J. F. Vallee B. L. (1986). Characteristic Ribonucleolytic Activity of Human Angiogenin. Biochemistry 25 (12), 3527–3532. 10.1021/bi00360a008 2424496

[B31] Shapiro R. Vallee B. L. (1987). Human Placental Ribonuclease Inhibitor Abolishes Both Angiogenic and Ribonucleolytic Activities of Angiogenin. Proc. Natl. Acad. Sci. 84 (8), 2238–2241. 10.1073/pnas.84.8.2238 3470787 PMC304624

[B32] Sheng J. Xu Z. (2016). Three Decades of Research on Angiogenin: a Review and Perspective. Acta Biochim. Biophys. Sin 48 (5), 399–410. 10.1093/abbs/gmv131 26705141 PMC4888354

[B33] Sheng J. Yu W. Gao X. Xu Z. Hu G.-F. (2014). Angiogenin Stimulates Ribosomal RNA Transcription by Epigenetic Activation of the Ribosomal DNA Promoter. J. Cel. Physiol. 229 (4), 521–529. 10.1002/jcp.24477 PMC4196338 24122807

[B34] Wei S. Gao X. Du J. Su J. Xu Z. (2011). Angiogenin Enhances Cell Migration by Regulating Stress Fiber Assembly and Focal Adhesion Dynamics. PLoS One 6 (12), e28797. 10.1371/journal.pone.0028797 22194915 PMC3237552

[B35] Weng C. Dong H. Chen G. Zhai Y. Bai R. Hu H. (2012). miR-409-3p Inhibits HT1080 Cell Proliferation, Vascularization and Metastasis by Targeting Angiogenin. Cancer Lett. 323 (2), 171–179. 10.1016/j.canlet.2012.04.010 22531314

[B36] Yeo K. J. Jee J.-G. Hwang E. Kim E.-H. Jeon Y. H. Cheong H.-K. (2017). Interaction between Human Angiogenin and the P53 TAD2 Domain and its Implication for Inhibitor Discovery. FEBS Lett. 591 (23), 3916–3925. 10.1002/1873-3468.12899 29105754

[B37] Yu D. Sun J. Weng Y. Luo L. Sheng J. Xu Z. (2021). Serum Angiogenin as a Potential Biomarker for Early Detection of Colorectal Adenomas and Colorectal Cancer. Anticancer Drugs 32 (7), 703–708. 10.1097/CAD.000000000000104 33661188

[B38] Yu W. Goncalves K. A. Li S. Kishikawa H. Sun G. Yang H. (2017). Plexin-B2 Mediates Physiologic and Pathologic Functions of Angiogenin. Cell 171 (4), 849–864. 10.1016/j.cell.2017.10.005 29100074 PMC5847377

[B39] Zhu J. Sheng J. Dong H. Kang L. Ang J. Xu Z. (2013). Phospholipid Scramblase 1 Functionally Interacts with Angiogenin and Regulates Angiogenin-Enhanced rRNA Transcription. Cell Physiol Biochem 32 (6), 1695–1706. 10.1159/000356604 24356419

