# Supplementary material for: Characterization and Function of the Interaction of Angiogenin With Alpha-Actinin 2
Source: Front Mol Biosci. 2022 Apr 8;9:837971. doi: 10.3389/fmolb.2022.837971 (PMC9033276; doi:10.3389/fmolb.2022.837971)
Supplement: Supplementary file 1 [file Table1.DOCX]

Supplementary Material

# Supplementary Table

Table.S1. Primers list

| Primer Name | Primer sequence | Restriction site |
| --- | --- | --- |
| *For ACTN2* |  |  |
| 284-F | 5’-ACGC*GTCGAC*CGAAGAATATGAGAGGCTAGCGAG | *Sal I* |
| 375-F | 5’-ACGC*GTCGAC*CTGGCAGAGGCTGGAGCAG | *Sal I* |
| 391-F | 5’-ACGC*GTCGAC*CAATGAGATTCGGAGACTGGAGC | *Sal I* |
| 398-F | 5’-ACGC*GTCGAC*CCGCTTGGAACACCTGGCTG | *Sal I* |
| 404-F | 5’-ACGC*GTCGAC*CGAGAAGTTCAGGCAGAAGGC | *Sal I* |
| 519-F | 5’-ACGC*GTCGAC*CCTGGAGTTTGCCAAGAGGGC | *Sal I* |
| 505-R | 5’-AAATAT*GCGGCCGC*TTACTCTAGGGCTTCTCTCCTCT | *Not I* |
| 518-R | 5’-AAATAT*GCGGCCGC*TTAGTGAAGCTGATCAATGGTTTCTAG | *Not I* |
| 620-R | 5’-AAATAT*GCGGCCGC*TTAGATGGGCACGAGTTGCTTCAC | *Not I* |
| 626-R | 5’-AAATAT*GCGGCCGC*TTACTGCAGGGATTGATCGCGGAT | *Not I* |
| 632-R | 5’-AAATAT*GCGGCCGC*TTACTGGCGAGCCAGCTCC | *Not I* |
| 655-R | 5’-AAATAT*GCGGCCGC*TTAGTTCTGGATCCAGGGCCC | *Not I* |
| 739-R | 5’-AAATAT*GCGGCCGC*TTACTCCACCTCATTGATGGTTCTG | *Not I* |
| GST-383-F | 5’-CG*GGATCC*AAGGGTTACGAGGAGTGGTTG | *BamH I* |
| GST-632-R | 5’-G*GAATTC*TTACTGGCGAGCCAGCTCCTC | *EcoR I* |
| cDNA-383-F | 5’-G*GAATTC*GCCACCATGGCAAAGGGTTACGAGGAGTGGTTG | *EcoR I* |
| cDNA-632-R | 5’-AAATAT*GCGGCCGC*TTACTGGCGAGCCAGCTCC | *Not I* |
| *For ANG* |  |  |
| 1-F | 5’-ACGC*GTCGAC*AGATAACTCCAGGTACACACACTT | *Sal I* |
| 42-F | 5’-ACGC*GTCGAC*CATCAACACATTTATTCATGGCAACAAG | *Sal I* |
| 63-F | 5’-ACGC*GTCGAC*CAACCCTCACAGAGAAAACCTAAGAAT | *Sal I* |
| 83-F | 5’-ACGC*GTCGAC*ACTACATGGAGGTTCCCCCTG | *Sal I* |
| 93-F | 5’-ACGC*GTCGAC*ACAGTACCGAGCCACAGCG | *Sal I* |
| 41-R | 5'-AAATAT*GCGGCCGC*TTAGTCTTTGCAGGGTGAGGTCAG | *Not I* |
| 62-R | 5'-AAATATGCGGCCGCTTATCCATTCTTGTTTTCACAGATGGCC | *Not I* |
| 82-R | 5'-AAATAT*GCGGCCGC*TTACTTGCAAGTGGTGACCTGGAAA | *Not I* |
| 93-R | 5'-AAATAT*GCGGCCGC*CTGGCATGGAGGCCAGG | *Not I* |
| 100-R | 5'-AAATAT*GCGGCCGC*GAACCCCGCTGTGGCTC | *Not I* |
| 105-R | 5'-AAATAT*GCGGCCGC*AACAACAACGTTTCTGAACCCCG | *Not I* |
| 110-R | 5'-AAATAT*GCGGCCGC*TTAGCCATTTTCACAAGCAACAACAAC | *Not I* |
| 123-R | 5'-AAATAT*GCGGCCGC*TTACGGACGACGGAAAATTGACTG | *Not I* |
| cDNA-sig-1-F | 5'-G*GAATTC*GCCACCATGGTGATGGGCCTGGGC | *EcoR I* |
| cDNA-123-R | 5'-AAATAT*GCGGCCGC*TTACGGACGACGGAAAATTGACTG | *Not I* |
| *For ANG mutation* |  |  |
| HGG84-86AAA | 5'-ACTTGCAAGCTAGCTGCAGCATCCCCCTGGCCT |  |
| SPW87-89AAG | 5'-GCTACATGGAGGTGCCGCCGGGCCTCCATGCCAG |  |
| PPCQ90-93AAAA | 5'-TTCCCCCTGGGCTGCAGCCGCGTACCGAGCCA |  |
| ATA96-98SSS | 5'-GCCAGTACCGATCCTCATCGGGGTTCAGAAAC |  |
| FNR100-102AAA | 5'-GCCACAGCGGGGGCCGCAGCCGTTGTTGTTGC |  |
| R95I | 5'-CCTCCATGCCAGTACATAGCCACAGCGGGG |  |
| R95A | 5'-CCTCCATGCCAGTACGCAGCCACAGCGGGG |  |
| R101A | 5'-GCCACAGCGGGGTTCGCAAACGTTGTTGTTGC |  |
| R101I | 5'-GCCACAGCGGGGTTCATAAACGTTGTTGTTGC |  |
| F100I | 5'-GCCACAGCGGGGATCAGAAACGTTGTTGTTGC |  |
